# Mortality among sexual and gender minority populations: A systematic review

**DOI:** 10.1371/journal.pone.0307688

**Published:** 2025-02-03

**Authors:** Corinne E. Zachry, Rory P. O’Brien, Kirsty A. Clark, Marissa L. Ding, John R. Blosnich

**Affiliations:** 1 Center for LGBTQ+ Health Equity, Suzanne Dworak-Peck School of Social Work, University of Southern California, Los Angeles, CA, United States of America; 2 Department of Medicine, Health and Society, Public Policy Studies, Vanderbilt University, Vanderbilt LGBTQ+ Policy Lab, Nashville, TN, United States of America; Virginia Tech Carilion Research Institute: Fralin Biomedical Research Institute at VTC, UNITED STATES OF AMERICA

## Abstract

Sexual and gender minority (SGM) populations experience elevated rates of negative health outcomes (e.g., suicidality) and social determinants (e.g., poverty), which have been associated with general population mortality risk. Despite evidence of disparities in threats to well-being, it remains unclear whether SGM individuals have greater risk of mortality. This systematic review synthesized evidence on mortality among studies that included information about SGM. Three independent coders examined 6,255 abstracts, full-text reviewed 107 articles, and determined that 38 met inclusion criteria: 1) contained a sexual orientation or gender identity (SOGI) measure; 2) focused on a mortality outcome; 3) provided SGM vs non-SGM (i.e., exclusively heterosexual and cisgender) or general population comparisons of mortality outcomes; 4) were peer-reviewed; and 5) were available in English. A search of included articles’ references yielded 5 additional studies (total n = 43). The authors used the NIH’s Quality Assessment Tool for Observational Cohort and Cross-Sectional Studies to assess included studies. Mortality outcomes included all-cause (n = 27), suicide/intentional self harm (n = 23), homicide (n = 7), and causes related to drug use (n = 3). Compared to non-SGM people, 14 studies (32.6%) supported higher mortality for SGM, 28 studies (65.1%) provided partial support of higher mortality for SGM (e.g., greater mortality from one cause but not another), one study (2.3%) found no evidence of higher mortality for SGM. There was considerable heterogeneity in operational definitions of SGM populations across studies. Although mixed, findings suggest elevated mortality for SGM versus non-SGM populations. Integrating SOGI measures into mortality surveillance would enhance understanding of disparities by standardizing data collection, thereby reducing heterogeneity and increasing capacity to aggregate results (e.g., meta-analyses).

## Introduction

Across the world, sexual and gender minority (SGM) individuals experience adverse social determinants of health and substantial disparities in many health risks [1, 2] For example, a recent review of reviews identified greater prevalence of interpersonal violence across studies from Africa, Asia, Australia, Europe, North and South Americas, and results of the World Health Organization World Mental Health Survey identified greater prevalence of substance use disorders amongst a global sample of sexual minority individuals. In the United States, the magnitude of these disparities has become clearer with emerging research following recent additions of self-reported sexual orientation and gender identity (SOGI) questions to federal health surveillance surveys [3–5]. For example, SGM populations more frequently report cigarette smoking, substance use, and suicidal ideation and attempt than do their non-SGM (i.e., identified as both exclusively heterosexual *and* exclusively cisgender) peers [6, 7].

In studies worldwide, minority stress has been used to explain the impacts of social stress experienced by people with stigmatized identities (e.g., SGM identities) [8–11]. Research on health disparities among SGM people has identified Minority Stress as a risk factor for many precursors to mortality, including suicidal ideation and attempts [12]. In addition to experiencing Minority Stress, which itself has been described as a social determinant of health for SGM people [13], SGM individuals are more likely than their non-SGM peers to report exposure to other harmful social determinants such as adverse childhood experiences [14], housing instability and homelessness [15], violent victimization [16], food insecurity [17], unemployment, and living in poverty [18]. Together, this research documents considerable adversity with clear implications for jeopardizing health, wellbeing, and longevity.

Despite evidence of reduced SGM health and wellbeing (e.g., psychopathology [9], physical health [19], worse self-rated health [20]), health equity research remains stymied by the lack of data about mortality. Globally, mortality information generally lacks in quality and coverage especially among countries in Africa and Asia, and to our knowledge, no government collects SOGI data in a standardized process at the time of death like other key demographics, such as age and sex [21, 22]. Consequently, it is unknown if mortality may be elevated for SGM populations.

The primary reasons to suspect greater mortality among SGM individuals hinge on evidence demonstrating that social adversity increases risk of mortality [23]. For example, in US studies that directly compare individuals experiencing homelessness to individuals with stable housing, risk of all-cause mortality ranges from 1.6 to over 3.0 times greater for homeless individuals [24–26]. SGM populations are more likely than their non-SGM peers to have experienced homelessness across the lifespan [27–29]. In another example, adverse childhood experiences are well-known predictors of premature mortality [30], with one US study estimating that experiencing >2 adverse childhood experiences increased risk of all-cause mortality by approximately 60% compared to people without adverse childhood experiences [31]. In the US, SGM populations are more likely to report adverse childhood experiences than their non-SGM peers [14, 32, 33], a finding that has been replicated in research in Asia and Europe [34]. For instance, Merrick et al. found that gay/lesbian adults reported an average of 2.2 adverse childhood experiences and bisexual adults reported an average of 3.1, compared to heterosexuals who reported an average of 1.6 [35]. Despite the substantial literature about greater burdens of social adversity faced by SGM populations and associations between social adversity and mortality risk, to date it is unclear whether the evidence about mortality, in aggregate, results in greater risk of death for SGM people.

Mortality data are both primary indicators of public health problems and necessary benchmarks for evaluating whether interventions to address public health problems are, in fact, reducing mortality or extending life. For example, inclusion of race in mortality surveillance made it possible to detect Black/African American women’s greater risk of maternal mortality in the US and to design public health policies and programs to address the increased risk [36]. Most mortality surveillance datasets do not collect SOGI data, which prevents such progress in public health knowledge and intervention for SGM individuals [37]. In one rare instance, the National Violent Death Reporting System (NVDRS) added sexual orientation and transgender status in 2013, but those data are not recorded systematically, with upwards of 80% of observations in the dataset missing information about sexual orientation and transgender status [38, 39]. Other studies in the US have attempted to assess SGM mortality using either self-reported survey data that were later paired with National Death Index (NDI) data [40, 41] or administrative data that permitted linkage to vital status information [42, 43]. Countries with national healthcare systems and marriage registers (e.g., Sweden, Denmark) have also attempted to estimate mortality through data linkages [42, 44]. However, findings from these studies on SGM mortality disparities are inconsistent and, given the limited assessment of SOGI information in mortality data, any one study is likely unable to depict SGM mortality disparities. However, in aggregate, existing studies may offer a more comprehensive assessment of the prevalence and magnitude of SGM disparities in mortality.

Thus, the current study sought to complete a systematic review of the literature on all-cause mortality among SGM individuals to explore whether studies of mortality indicate greater mortality rates or risk among SGM people in relation to non-SGM or general population comparators.

## Methods

### Inclusion/exclusion criteria

Included studies were 1) written or available in English; 2) published after the inception of the National Center for Health Statistics in 1960 for the collection and dissemination of mortality data [45]; 3) peer-reviewed; 4) measured sexual orientation and/or gender identity (inclusive of lesbian, gay, bisexual, transgender, or queer [LGBTQ+] sexual orientations and gender identities); 5) reported a mortality rate outcome of any cause; and 6) reported disaggregated mortality outcomes for SGM and a non-SGM comparison group (including either cisgender vs transgender, gay/bi/lesbian vs heterosexual, or SGM vs non-SGM). We excluded studies that did not meet inclusion criteria or 1) utilized exclusively qualitative methods; or 2) had a sample which consisted *solely* of people living with HIV or AIDS, given that this health crisis is well-known to have greatly skewed mortality among men who have sex with men and transgender women [46, 47]. However, HIV/AIDS was still included as a possible cause of death among samples with mixed HIV status. There were no restrictions or exclusions based upon geographical origin of study data and/or study participants. Inclusion and exclusion criteria are summarized in Table 1.

**Table 1:** Summary of inclusion and exclusion criteria.

Inclusion	Exclusion
1) Written or available in English	1) Utilized exclusively qualitative methods
2) Published after 1960	2) Sample consisted *solely* of people living with HIV or AIDS
3) Peer-reviewed	
4) Measured sexual orientation and/or gender identity	
5) Reported a mortality rate outcome of any cause	
6) Reported disaggregated mortality outcomes for SGM and a non-SGM comparison group	

### Search process

The initial systematic search of the literature pertaining to mortality of SGM people was conducted on June 29, 2021. To ensure up-to-date findings, an additional search was conducted on July 18, 2023. Both searches used seven databases; APA (including PsycArticles, PsycBooks, PsycInfo), Cumulative Index to Nursing and Allied Health Literature (CINAHL) Complete, Health Policy Reference Center, LGBTQ+ Source, Social Sciences Full Text, Health Source, MEDLINE and PubMed. In consultation with a systematic review librarian, database-specific thesauruses were used to generate core search terms relevant to sexual orientation (e.g., lesbians, gay men, bisexuals, sexual minorities), gender identity (e.g., transgender people, gender nonconformity), and mortality (e.g., death, suicide, homicide). The search was filtered by date (1960-present), language (English), and peer-review status. The review protocol and other materials are available upon request. The complete list of final search parameters is available in S1 Table.

### Study team

The study team is comprised of five researchers, inclusive of faculty, doctoral students and an undergraduate student with research backgrounds spanning social work, public health, policy, emergency medicine, health service delivery and psychology. A majority of the members of the research team identify as sexual minority and/or gender diverse.

### Coding and extraction

**Fig 1:**
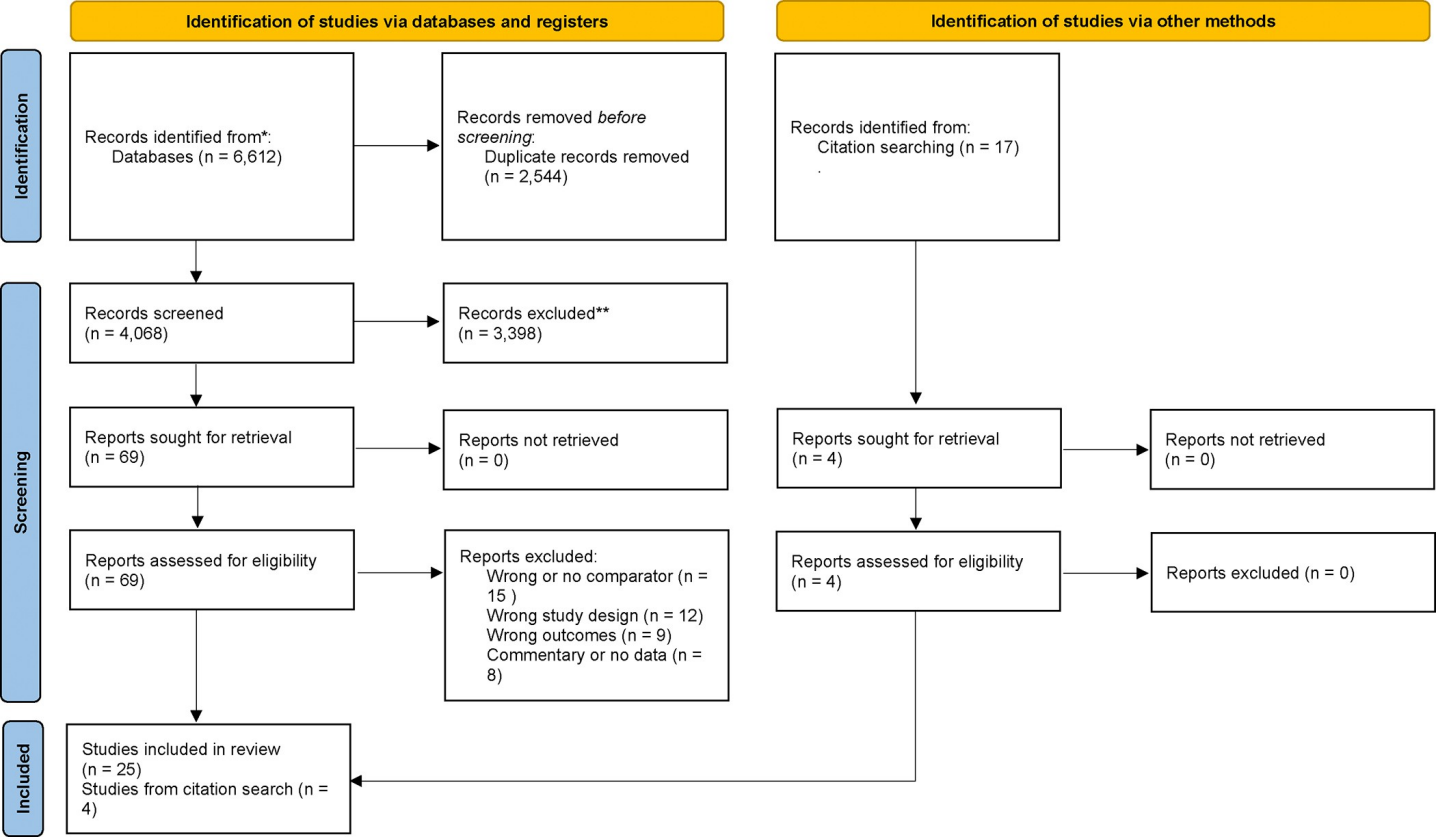
PRISMA flow diagram, initial search.

**Fig 2:**
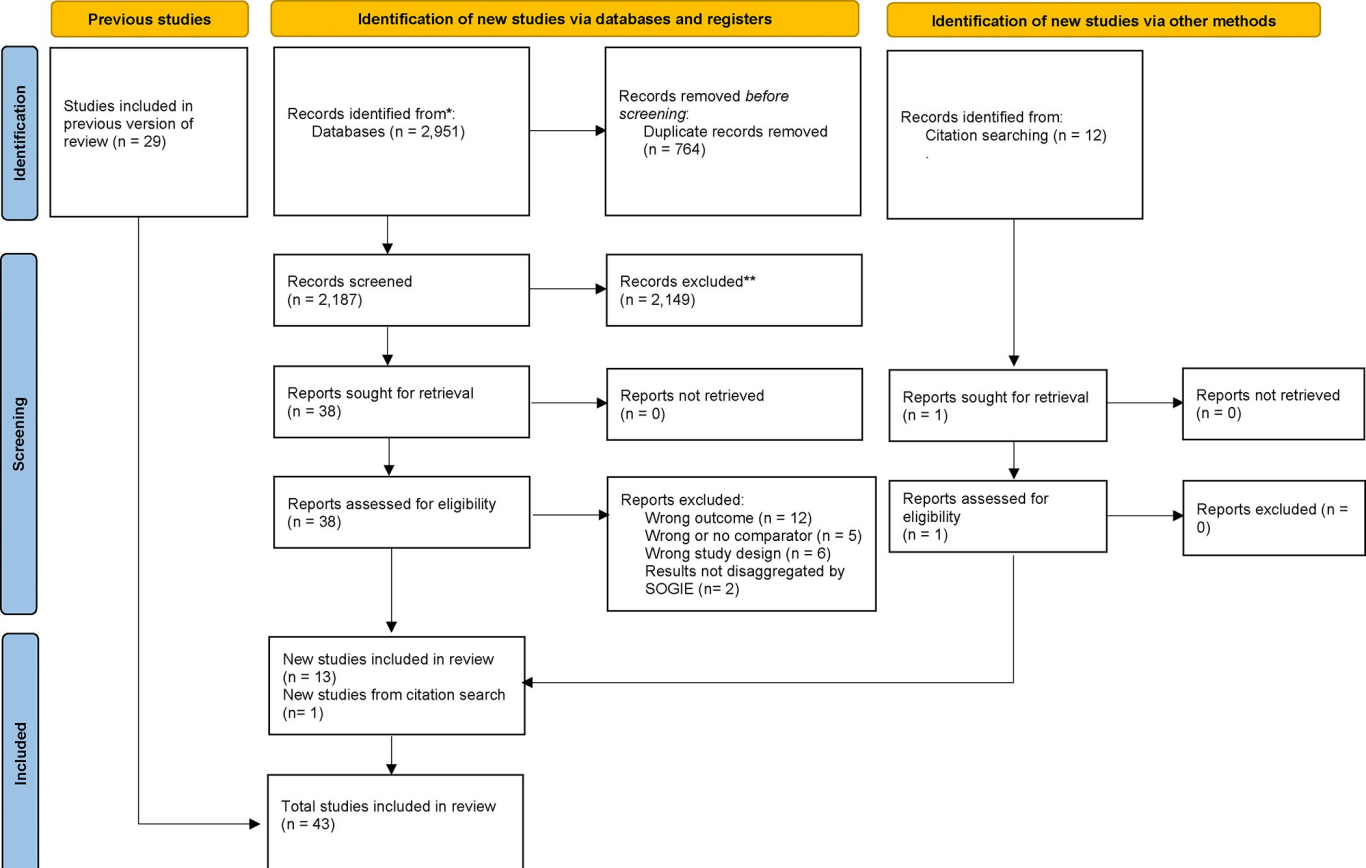
PRISMA flow diagram, updated search.

The first and second author independently conducted the database searches using the identified key terms. Search results were compared and assessed for inconsistencies prior to being uploaded into the systematic review program Covidence, which mechanizes and documents the processes outlined in the PRISMA protocol [48], including removing duplicate records, screening, and extracting data. Utilizing PRISMA guidelines (Figs 1 and 2), titles and abstracts of 6,255 records were screened and dually coded by the first and second authors in the initial title and abstract screening stage. Articles were included in the full-text review if they appeared to meet inclusion criteria directly (i.e., specifying a mortality outcome and SGM participants) or if they specified a mortality outcome while being ambiguous about SOGI of participants (e.g., abstracts referring to “gender differences” broadly without specifying whether a binary measure of gender was used; or those referring ambiguously to “demographic factors”). Of those records, 107 full texts were reviewed and dually coded by the first and second authors for possible inclusion, of which 38 were included for this study.

The first, second, and senior authors conducted an ancestral search by reviewing reference sections from these 38 included articles to identify any potentially relevant titles. Twenty-nine potentially relevant titles were identified for abstract and title screening, of which 5 full text records were evaluated and 5 records included, bringing the total number of included studies to 43. Discrepancies in coding in any stage were resolved by the senior author. In the title and abstract screening stage, inter-rater agreement was 96.6%. Inter-rater agreement for the full text review stage was 85.1%.

The first, second, and senior authors independently extracted the following key information about each included study: country of origin, research questions/aims, hypotheses, data sources, sampling frames, population description, inclusion criteria, exclusion criteria, characteristics of comparison groups, whether the comparison was direct or indirect, study denominator or unit of analysis (i.e., sample size or person years), mortality metric, statistical methods and key findings. A summary of extracted data is presented in Table 2, and full detail on extracted data can be found in Table 3. Due to the heterogeneity of data sources, populations, predictors and outcomes among the included studies, the authors were not able to conduct a meta-analytic review. For example, included studies reported wide-ranging causes of death (e.g., accidents, intentional self-harm/suicide, influenza and pneumonia, breast cancer) for which risk and survival are not comparable. Moreover, samples of SGM people in included studies were often comprised of individuals with characteristics that limit the generalizability of findings on mortality risk; for example, despite shared identities under the SGM umbrella, mortality rates among modern-day sexual minority United States military veterans [49], transgender people who have received gender affirming hormone therapy in the Netherlands [50], people who had been diagnosed with homosexuality as a psychiatric illness in the 1960s [51], people who use injection drugs [52] and patients in primary care clinics in the United Kingdom [53] cannot be assumed to be directly comparable due to potential differences in life experience that contribute to mortality risk.

**Table 2:** Summary of literature review findings.

Cause of death	SGM subgroup	Study	Mortality findings
**Accidents**	Sexual minority people	Lynch et al., 2020	**⇑**
		Salway et al., 2022	**⇑**
	Gender minority people	Asscheman, Gooren & Eklund, 1989	**∅**
		van Kesteren, Asscheman, Megens & Gooren, 1997	!
**All cause**	Sexual minority people	Cochran & Mays, 2011	**⇑**
		Cochran & Mays, 2012	**∅**
		Cochran & Mays, 2015	**∅**
		Cochran, Bjorkenstam & Mays, 2016	**⇑**
		Davis et al., 2017	≈
		Everett et al., 2021	**⇑**
		Frisch & Brønnum-Hansen, 2009	**⇑**
		Frisch & Simonsen, 2013	**⇑**
		Koblin et al., 1992	**⇑**
		Laughney & Eliason, 2022	≈
		Lehavot et al., 2016	≈
		Lindstrom & Rosvall, 2020	≈
		Lynch et al., 2020	**⇑**
		Martin, Cloninger, Guze & Clayton, 1985	**⇑**
		Passaro et al., 2019	**⇑**
		Salway et al, 2022	**⇑**
	Gender minority people	Asscheman et al., 2011	≈
		Asscheman, Gooren & Eklund, 1989	≈
		Blosnich et al., 2014	≈
		Boyer et al., 2021	**⇓**
		deBlok et al., 2021	≈
		Dhejne et al., 2011	**⇑**
		Erlangsen et al., 2023	**⇑**
		Hughes et al., 2022a	≈
		Hughes et al., 2022b	**⇑**
		Jackson et al., 2023	≈
		van Kesteren, Asscheman, Megens & Gooren, 1997	**∅**
**Alzheimer’s disease**	Sexual minority people	Lynch et al., 2020	**⇑**
**Cardiovascular disease**	Sexual minority people	Frisch & Simonsen, 2013	≈
		Lehavot et al., 2016	**∅**
		Lynch et al., 2020	**⇑**
		Salway et al., 2022	**⇑**
	Gender minority people	Asscheman et al., 2011	≈
		Asscheman, Gooren & Eklund, 1989	**∅**
		deBlok et al., 2021	≈
		Dhejne et al., 2011	**⇑**
		Jackson et al., 2023	**∅**
		van Kesteren, Asscheman, Megens & Gooren, 1997	**∅**
**Cancer**	Sexual minority people	Cochran & Mays, 2012	**⇑**
		Cochran & Mays, 2015	**∅**
		Frisch & Simonsen, 2013	≈
		Lehavot et al., 2016	**⇑**
		Lynch et al., 2020	**⇑**
		Moore et al., 2023	≈
		Salway et al., 2022	**∅**
	Gender minority people	Asscheman et al., 2011	**⇑**
		deBlok et al., 2021	≈
		van Kesteren, Asscheman, Megens & Gooren, 1997	**∅**
**Digestive disease**	Gender minority people	Asscheman et al., 2011	**∅**
		Blosnich et al., 2014	**⇑**
		Jackson et al., 2023	**∅**
		van Kesteren, Asscheman, Megens & Gooren, 1997	**∅**
**Drug-use related**	Sexual minority people	O’Driscoll et al., 2001	≈
		Lynch et al., 2022	**⇑**
	Gender minority people	Asscheman et al., 2011	**⇑**
**Endocrine disease**	Sexual minority people	Lynch et al., 2020	**⇑**
	Gender minority people	Asscheman et al., 2011	**∅**
		Jackson et al., 2023	**∅**
**Genitourinary disease**	Gender minority people	Asscheman et al., 2011	**∅**
**HIV/AIDS related**	Sexual minority people	Cochran & Mays, 2011	**⇑**
		Cochran & Mays, 2015	**∅**
		Frisch & Simonsen, 2013	≈
		Koblin et al., 1992	**⇑**
		Salway et al., 2022	!
	Gender minority people	Asscheman et al., 2011	≈
		deBlok et al., 2021	**⇑**
		van Kesteren, Asscheman, Megens & Gooren, 1997	**⇑**
**Homicide**	Sexual minority people	Anderson et al., 2023	**⇑**
		Mize & Shackelford, 2008	≈
		Ndimbie et al., 1994	!
	Gender minority people	Anderson et al., 2023	**∅**
		Asscheman, Gooren & Eklund, 1989	**∅**
		Boyer et al., 2021	**⇑**
		Dinno, 2017	**⇓**
		van Kesteren, Asscheman, Megens & Gooren, 1997	**∅**
**Infectious and parasitic disease**	Gender minority people	Blosnich et al., 2014	**⇑**
		deBlok et al., 2021	≈
		Jackson et al., 2023	**∅**
**Influenza and pneumonia**	Sexual minority people	Lynch et al., 2020	**⇑**
**Intentional self-harm/Suicide**	Sexual minority people	Alvarez-Hernandez & Mowbray, 2023	**⇑**
		Bjorkenstam et al., 2016	≈
		Cochran & Mays, 2015	≈
		Erlangsen et al., 2020	**⇑**
		Feigelman, Ploderl, Rosen & Cerel, 2019	≈
		Frisch & Brønnum-Hansen, 2009	≈
		Frisch & Simonsen, 2013	≈
		Lynch et al., 2020	**⇑**
		Mathy, Cochran, Olsen & Mays, 2011	**⇑**
		Ndimbie et al., 1994	**∅**
		Ream, 2019	**⇑**
		Salway et al., 2022	**∅**
	Gender minority people	Asscheman et al., 2011	≈
		Asscheman, Gooren & Eklund, 1989	**⇑**
		Biggs, 2022	**⇑**
		Blosnich et al., 2014	**⇑**
		Blosnich et al., 2021	**⇑**
		Boyer et al., 2021	**⇑**
		deBlok et al., 2021	≈
		Dhejne et al., 2011	**⇑**
		Erlangsen et al., 2023	**⇑**
		Ream, 2019	**⇑**
		van Kesteren, Asscheman, Megens & Gooren, 1997	**⇑**
		Wiepjes et al., 2020	**⇑**
**Kidney disease**	Sexual minority people	Lynch et al., 2020	**⇑**
**Mental and behavioral disorders**	Gender minority people	Jackson et al., 2023	**∅**
**Neoplasm**	Gender minority people	Asscheman et al., 2011	**∅**
		Dhejne et al., 2011	**⇑**
		Jackson et al., 2023	**∅**
**Nervous system disease**	Gender minority people	Asscheman et al., 2011	**∅**
		Blosnich et al., 2014	**⇓**
		Jackson et al., 2023	**∅**
**Pancreatitis**	Gender minority people	Asscheman, Gooren & Eklund, 1989	**∅**
		van Kesteren, Asscheman, Megens & Gooren, 1997	**∅**
**Respiratory disease**	Sexual minority people	Frisch & Simonsen, 2013	≈
		Lynch et al., 2020	**⇑**
	Gender minority people	Asscheman et al., 2011	**∅**
		Asscheman, Gooren & Eklund, 1989	**∅**
		Jackson et al., 2023	**∅**
		van Kesteren, Asscheman, Megens & Gooren, 1997	**∅**
**Stroke**	Sexual minority people	Lynch et al., 2020	**⇑**
	Gender minority people	Asscheman et al., 2011	**∅**
**Unknown cause of death**	Gender minority people	Asscheman et al., 2011	≈
		Asscheman, Gooren & Eklund, 1989	**⇑**
		van Kesteren, Asscheman, Megens & Gooren, 1997	**∅**
**Another cause of death**	Sexual minority people	Cochran & Mays, 2011	**∅**
		Cochran & Mays, 2015	**∅**
		Frisch & Simonsen, 2013	**⇑**
		Ndimbie et al., 1994	!
	Gender minority people	Blosnich et al., 2014	**∅**
		deBlok et al., 2021	≈
		Jackson et al., 2023	≈

**⇑** denotes consistently elevated morality for a particular cause/group; ≈ denotes some evidence of elevated mortality for a particular cause/group; **∅** denotes no evidence of elevated mortality for a particular cause/group**; ⇓** denotes evidence of lower mortality for a particular cause/group;! indicates insufficient information for analysis

**Table 3:** Comprehensive literature review overview matrix.

Study	Data source(s) and Country	Study population	Comparison group (direct or indirect)	Study denominator (*n* or person years)	Causes of death examined	Key findings on mortality for SGM*	QA Rating
**Studies reporting consistently elevated mortality among SGM**							
Alvarez-Hernandez & Mowbray, 2023	National Violent Death Reporting System (NVDRS), United States	Hispanic individuals whose deaths were recorded in NVDRS in 32 US states; 2012–2016	Heterosexual Hispanic individuals in NVDRS (direct)	**•** Total *n* = 1,132 **•** 5.1% LGB	**•** Suicide	**⇑ suicide mortality for LGB** (OR = 2.23 [1.12–4.45)	Good
Biggs, 2022	Suicides reported to Tavistock Board meetings, National Health Service, United Kingdom	Total patients referred to Gender Identity Development Service (GIDS); 2010–2020	Overall population of adolescents aged 14 to 17 in the United Kingdom (indirect)	**•** Total *n* = 15,032 **•** Patient years = 30,080 **•** Deaths *n* = 4	**•** Suicide	**⇑ GIDS patient suicide death rate** was 13/100,000; rate for overall population of adolescents 14–17 was 2.7/100,00; authors estimate GIDS patients had 5.5 times greater rate of suicide	Fair
Dhejne et al., 2011	Linkage of several Swedish national registers: Hospital Discharge, Total Population, Medical Birth, Education, Cause of Death, Crime, Sweden	Individuals diagnosed with gender identity disorder & whose gender changed in register; 1973–2003	Randomly selected controls with consistent sex designation & no gender identity disorder diagnosis (direct)	**•** Total *n* = 3,564; **•** People with gender changed *n* = 324; **•** Control *n* = 3,240	**•** All-cause **•** Suicide **•** Cardiovascular disease (CVD) **•** Neoplasm	**Among individuals whose gender changed:** **⇑ all-cause (**aHR = 2.8 [1.8–4.3]) **⇑ suicide** (aHR = 19.1 [5.8–62.9]) **⇑ CVD** (aHR = 2.5 [1.2–5.3]) **⇑ neoplasm** (aHR = 2.1 [1.0–4.6])	Good
Erlangsen et al., 2020	Danish Centralized Civil Register and Swedish Total Population Register, Danish and Swedish Cause of Death Registers, Denmark and Sweden	People who married same-sex partners; 1989–2016	People who married different-sex partners between 1989–2016 (direct)	**•** Total *n* = 3,947,266 **•** Same-sex married *n* = 28,649 **•** Different-sex married *n* = 3,918,617	**•** Suicide	**Among people who entered same-sex marriages:** **⇑ suicide** (IRR = 2.3, [1.9–2.8]) **⇑ suicide** (females IRR = 2.7 [1.6–2.5]; males (IRR = 2.1 [1.7–2.7]), **⇑ suicide among older adults** (IRR = 2.8 [1.3–6.1])	Good
Erlangsen et al., 2023	Danish Civil Registration System, Denmark	Transgender people living in Denmark, aged 15 years or older; 1980–2021	Non-transgender individuals’ mortality rates (direct)	**•** Total *n* = 6,657,456 **•** Total person years = 171,023,873 **•** Transgender people = 3,759	**•** All-cause **•** Suicide	**Among transgender people:** **⇑ suicide mortality** (aIRR = 3.5 [2.0–6.3]) **⇑ non-suicide mortality**(aIRR = 1.9 [1.6–2.2]) **⇑ all-cause mortality** (aIRR = 2.0 [1.7–2.4])	Good
Everett et al., 2021	The Chicago Health and Life Experiences of Women (CHLEW); National Death Index; next-of-kin reports, United States	Sexual minority women who participated in CHLEW	Life tables from Centers for Disease Control and Prevention for deaths expected in a general U.S. population of women with same age distribution and length of follow-up as those in the CHLEW sample (indirect)	• Total *n* = 775 **•** Deaths = 49	**•** All-cause	**⇑ all-cause mortality prevalence for sexual minority women** (6.32%) vs. expected mortality prevalence for general population of U.S. women (5.27%)	Good
Frisch & Brønnum-Hansen, 2009	Danish Civil Registration System, Denmark	People who were in a same-sex marriage for at least 1 year; 1996–2011	Danish general population mortality rate (indirect)	**•** Total *n* = 8,333 **•** Deaths = 618 **•** Person years = 60,316	**•** All-cause	**Among those in a same-sex marriage:** **⇑ overall excess death rate for men** (5.92/1000) **and women** (1.15/1000) **⇑ overall rate** for men (SMR = 1.78 [1.63–1.94]) and women (SMR = 1.34 [1.09–1.63]) **⇑ overall rate highest in the 1–3 year period after marriage** for men (SMR = 2.65 [2.28–3.06]) and women (SMR = 1.91 [1.36–2.60])	Good
Koblin et al., 1992	Epidemiological study, NYC Death Registry, United States	NYC MSM in two studies of hepatitis B virus infection; late 1970s	Mortality rate of white men in the US aged 25–64 (indirect)	**•** Total *n* = 8,906 **•** Persons with cause of death data = 968	**•** All-cause **•** HIV/AIDS	**⇑ MSM** (SMR = 3.7 [3.4–3.9]) 74.7% of deaths among MSM due to HIV-1/AIDS	Fair
Lynch et al., 2020	VHA Corporate Data Warehouse, National Death Index, United States	Veterans within the VHA outpatient care system with documented sexual minority identity between 2000–2017	Mortality rates in the US population and the general VHA population (indirect)	**•** Total *n* = 96,893 **•** Total deaths = 12,591	**•** All-cause **•** Suicide **•** Cancer **•** Heart disease **•** Accidents **•** Respiratory disease **•** Stroke **•** Diabetes **•** Alzheimer’s disease **•** Influenza and pneumonia **•** Kidney disease	**Among sexual minority veterans:** **⇑ suicide** (SMR = 4.50 [4.13–4.99]) compared to US population rate **⇑ suicide** (HR = 1.59 [1.55–1.64]) compared to non-sexual minority veterans **⇑ cancer** (SMR = 3.35 [3.32–3.47]) compared to US population **⇑ heart disease** (SMR = 3.78 [3.64–3.93]) compared to US population **⇑ accident** (SMR = 2.82 [2.64–3.02]) compared to US population **⇑ chronic lower respiratory disease** (SMR = 3.20 [2.96–3.45]) compared to US population **⇑ stroke** (SMR = 2.50 [2.27–2.73]) compared to US population **⇑ diabetes** (SMR = 3.37 [3.06–3.71]) compared to US population **⇑ Alzheimer’s disease** (SMR = 5.55 [4.96–6.19]) compared to US population **⇑ influenza and pneumonia** (SMR = 2.86 [2.48–3.28]) compared to US population **⇑ kidney disease** (SMR = 2.27 [1.93–2.66]) compared to US population	Good
Lynch et al., 2022	Veterans Health Administration (VHA) electronic health records; National Death Index, United States	Patients receiving VHA health care	5,300,521 non-LGB veterans (direct)	**•** Total *n* = 537,661 **•** LGB *n* = 6,267	**•** Alcohol-attributable death	**Among all LGB veterans**: **⇑ overall alcohol-attributable mortality** (RR = 1.57 [1.48–1.66]) **⇑ chronic alcohol-attributable causes of mortality** (RR = 1.52 [1.40–1.64]) **⇑ acute alcohol-attributable causes of mortality** (RR = 1.64 [1.50–1.78]) **Among male LGB veterans:** **⇑ overall alcohol-attributable mortality** (RR = 1.82 [1.72–1.93]) **⇑ chronic alcohol-attributable causes of mortality** (RR = 1.70 [1.57–1.83]) **⇑ acute alcohol-attributable causes of mortality** (RR = 1.97 [1.80–2.14]) **Among female LGB veterans:** **⇑ overall alcohol-attributable mortality** (RR = 3.01 [2.66–3.35]) **⇑ chronic alcohol-attributable causes of mortality** (RR = 4.14 [3.57–4.72]) **⇑ acute alcohol-attributable causes of mortality** (RR = 2.34 [1.96–2.73])	Good
Martin, Cloninger, Guze & Clayton, 1985	Survey and interviews of patients from Jul 1, 1967—Nov 1, 1969, United States	Washington University in St Louis Clinic patients with psychiatric diagnosis of homosexuality	Age-, sex-, and race-specific expectations derived from 1970 death rates for Missouri (indirect)	**•** Total *n* = 494 **•** Total deaths = 43	**•** All-cause	**Among patients with index psychiatric diagnosis of homosexuality:****⇑ unnatural death** (SMR = 16.67 [95% CI not provided], *p* < 0.01)no natural deaths**! no natural deaths**	Poor
Passaro et al., 2019	Data collected from gay and bisexual men (GBM) recruited in LA through media outlets & community centers, 1998–2000, United States	GBM who took part in an RCT for treatment of meth use/addiction	Populations reported in CDC WONDER, matched by geography, age, & sex (indirect)	**•** Total *n* = 191 **•** Total deaths = 24	**•** All-cause	**⇑ all-cause for GBM with methamphetamine dependence** (10 year SMR = 3.95 [2.89–5.01]; 20 year SMR = 3.39 [2.69–4.09]); mortality among GBM sample did not significantly differ based on HIV status (HIV+ CMR = 5.2/1,000 PY; HIV- CMR = 2.3/1,000PY; *p* = 0.22)	Fair
Ream, 2019	NVDRS 2013–2015, United States	LGBT youth and young adults who died by suicide	Youth and young adults who died by suicide & are not identified as LGBT (direct); comparison of LGBT youth percent of sample to LGBT percent in general population (indirect)	**•** *n* = 2,209	**•** Suicide	**⇑ suicide prevalence** 8–24% **among LGBT youth and young adults**, who comprise 4.5% of the general population	Poor
Wiepjes et al., 2020	Medical charts for visitors to Center of Expertise on Gender Dysphoria of the Amsterdam UMC; National Civil Record Registry; hospital registration system, psychological, & medical files for cause of death, Netherlands	Clinically referred transgender people	Dutch population overall suicide rate for 2013–2017 (indirect)	**•** Total *n* = 8,263 **•** Total deaths by suicide = 49	**•** Suicide	**⇑ mean number of suicides in transgender population** years 2013–2017 (overall 40/100,000 PY, transgender women 43/100,000 PY, transgender men 34/100,000 PY, Dutch population overall 11/100,000 PY) **⇑ suicide risk for transgender women**, compared to transgender men (HR = 2.26 [1.06–4.82])	Good
**Studies reporting some evidence of elevated mortality among SGM**							
Anderson et al., 2023	NVDRS, 2003–2017, United States	Homicide deaths among women in NVDRS-participating states	Non-SGM women decedents of IPH (direct)	**•** Total *n* = 18,702 **•** SGM homicide deaths *n* = 238 **•** SGM IPH homicide deaths = 123	**•** Intimate partner homicide (IPH)	**⇑ IPH mortality for sexual minority women** across racial groups; Black/African American (OR = 7.84 [3.65–16.88]), White (OR = 2.30 [1.03–5.17]), and Other race (OR = 12.83 [2.83–58.29) ∅ **IPH mortality for transgender women** across racial groups; Black/African American (OR = 0.97 [0.43–2.24]), White (OR = 1.12 [0.17–7.24]), and Other race (OR = 3.03 [0.30–30.20])	Good
Asscheman, Gooren & Eklund, 1989	Medical files from the authors’ outpatient department, Netherlands	Individuals who have had cross-sex hormone treatment	Dutch general population mortality rate (indirect)	**•** Total *n* = 425 **•** Total deaths = 12	**•** All-cause **•** Suicide **•** Accidental drowning **•** Homicide **•** Myocardial infarction **•** COPD **•** Pancreatitis **•** Unknown cause	**⇑ overall mortality of transgender patients** 2.5x to 9.0x expected mortality based on general population rate **⇑ suicide for transgender women** greater than expected rate (3 observed [0.63–8.80]; 0.21 expected) **⇑ unknown cause for transgender women** greater than expected rate (3 observed [0.63–8.80], 0.10 expected) ∅ **rates of all other causes of death not significantly different** from expected rates	Good
Asscheman et al, 2011	Outpatient data, Dutch National Civil Record Registry, general population mortality, Netherlands	Individuals who have had cross-sex hormone treatment starting before July 1, 1997 and followed up for at least one year	General Dutch population mortality rate (indirect)	**•** Total *n* = 1,331 **•** Total deaths = 134	**•** All-cause **•** Suicide **•** Neoplasm **•** Heart disease **•** Cerebro-vascular accident **•** AIDS **•** Endocrine /diabetes **•** Respiratory disease **•** Digestive disease **•** Genitourinary disease **•** Nervous system disease **•** Illicit drug use **•** Unknown cause	**Among transgender women:** **⇑ all-cause** (SMR = 1.51 [1.47–1.55]) **⇑ lung cancer** (SMR = 1.35 [1.14–1.58]) **⇑ hematological cancer** (SMR = 2.58 [1.97–3.30]) **⇑ heart disease** (SMR = 1.64 [1.43–1.87]) **⇑ AIDS** (SMR = 30.20 [1.47–1.55]) **⇑ illicit drug use** (SMR = 13.20 [9.70–17.6]) **⇑ suicide** (SMR = 5.70 [4.93–6.54]) **⇑ unknown cause** (SMR = 4.00 [3.53–4.51]) **∅ all-cause age 65+** (SMR = 0.95 [0.86–1.06]) **∅ other neoplasm** (total SMR = 0.98 [0.88–1.08]; digestive tract SMR = 0.42 [0.28–0.60]; brain SMR = 1.59 [0.95–2.46]; “other” SMR = 0.79 [0.57–1.07]) **∅ cerebrovascular accident** (SMR = 1.26 [0.93–2.64]) **∅ endocrine/diabetes** (SMR = 0.85 [0.41–1.32]) **∅ respiratory disease** (SMR = 0.85 [0.61–1.14]) **∅ digestive disease** (SMR = 1.01 [0.68–1.45]) **∅ genitourinary disease** (SMR = 1.01 [0.68–1.45]) **! no nervous system disease deaths observed** Among **transgender men:** **⇑ illicit drug use** (SMR = 25.00 [6.00–32.5]) **∅ all-cause** (SMR = 1.12 [0.89–1.59]) **∅ all neoplasm** (total SMR = 0.99 [0.65–1.44]; lung SMR = 1.06 [0.26–3.19]; digestive tract SMR = 0.42 [0.28–0.60]; hematological SMR = 2.86 [0.69–8.57]; “other” SMR = 0.77 [0.25–1.77]) **∅ heart disease** (SMR = 1.19 [0.39–2.74]) **∅ digestive disease** (SMR = 2.56 [0.62–7.69]) **∅ nervous system disease** (SMR = 3.57 [0.86–10.7]) **∅ suicide** (SMR = 2.22 [0.53–6.18]) **∅ unknown cause** (SMR = 2.08 [0.69–4.79]) **! no deaths observed by brain neoplasm, cerebrovascular accidents, AIDS, endocrine/diabetes, respiratory system, or genitourinary system**	Good
Bjorkenstam et al., 2016	Swedish Longitudinal Integration Database for Health Insurance and Labor Market Studies (LISA), National Patient Register, Causes of Death Register, Sweden	Residents who were newly partnered or married between January 1, 1996 and December 31, 2009	Residents in different sex marriages or partnerships (direct)	• Total *n* = 1,188,181 • Deaths = 865	**•** Suicide	**⇑ suicide among same-sex married individuals** (IRR = 2.7 [1.5–4.8]) **⇑ suicide among same-sex married men** (IRR = 2.8 [1.5–5.5]), **partially attenuated** by adjusting for HIV status (aIRR = 2.3 [1.2–4.8]) **∅ suicide among same-sex married women** (IRR = 2.5 [0.8–7.7])	Good
Blosnich et al., 2014	VA National Patient Care Database, National Death Index mortality data, United States	VHA users with an ICD-9-CM transgender-related diagnosis	U.S. general population & non-transgender VHA population rates (indirect)	• Total *n* = 5,117 • Deaths = 309	• All-cause **•** Suicide • Other leading causes of death	**Among transgender veterans:** **⇑ suicide** (crude rate 82.5/100,000 person years for transgender veterans vs. 37.7/100,000 person years for VHA population) **⇑ digestive system disease** (6.8% of deaths for transgender cohort vs. 3.6% in US population) **⇑ infectious and parasitic disease** (6^th^ leading cause of death for transgender veterans cohort vs. 9^th^ leading cause in US population) **⇓ diseases of the nervous system** (8^th^ leading cause of death among transgender veterans cohort vs. 5^th^ leading cause in US population) **∅ patterns of other mortality causes**	Fair
Blosnich et al., 2021	VHA EHR patient data 1999–2016, cross-referenced with National Death Index data up to Dec 31 2016, United States	Transgender veterans, categorized based on ICD codes related to gender dysphoria, who were patients of the VHA between 1999–2016	3 non-transgender VHA patients randomly matched to a transgender patient at same VHA facility within 10 day window (direct)	• Total *n* = 35,905 • Deaths = 8,866	**•** Suicide	**Among transgender patients:** **⇑ suicide prevalence** (0.81% for transgender patients vs. 0.26% for non-transgender patients, *p* < 0.01) **⇑ suicide, firearm** (aHR = 1.83 [1.14–2.94]) **⇑ suicide, poisoning** (aHR = 3.08 [1.33–7.14]) **∅ suicide, hanging/suffocation** (aHR = 1.21 [0.52–3.27]) **∅ suicide, other unspecified means** (aHR = 1.05 [0.17–6.53])	Good
Boyer et al., 2021	VHA EHR patient data 1999–2016, cross-referenced with National Death Index data up to Dec 31 2016, United States	Transgender veterans, categorized based on ICD codes related to gender identity, who were patients of the VHA between 1999–2016	3 Non-transgender VHA patients randomly matched to a transgender patient’s ICD diagnosis at the same VHA facility (direct)	• Total *n* = 32,441	**•** All-cause **•** Suicide **•** Homicide	**Among transgender veterans:** **⇑ suicide** (aHR = 2.77 [1.88–4.09]) **⇑ suicide age 18–39** (aHR = 3.35 [1.30–8.60]) **⇑ suicide age 65+** (aHR = 9.48 [3.88–23.19]) **⇓ all-cause (**aHR = 0.90 [0.84–0.97]) **∅ all-cause age 18–39** (aHR = 1.07 [0.73–1.58]) **⇓ all-cause age 40–64** (aHR = 0.78 [0.72–0.86]) **⇑ all-cause age 65+** (aHR = 1.17 [1.03–1.33]) **⇑ homicide** (HR = 3.67 [1.51–8.91])	Good
Cochran & Mays, 2011	NHANES III 1988–1994 and National Death Index up to Dec 31 2006, United States	Men in the United States	Male participants with "female partners only" & "no sexual partners" (direct)	• Total *n* = 5,574 • Deaths = 515	• All-cause • HIV-related • Causes other than HIV	**Among men with any male sexual partners:** **⇑ HIV-related** (aHR = 157.37 [59.77–414.32]) **⇑ all-cause** (aHR = 3.59 [1.91–6.74]) **∅ non-HIV-related** (aHR = 1.37 [0.58–3.21])	Good
Cochran & Mays, 2012	National Health Interview Survey, 1997–2003, United States	Married and cohabiting women	Married or cohabiting women with different-sex relationship partner (direct)	• Total *n* = 136,867 • Deaths = 4,396	• All-cause • Breast cancer	**Among sexual minority women:** **∅ all-cause** (aHR = 1.23 [0.66–2.32]) **⇑ breast cancer** (aHR = 3.24 [1.01–10.37])	Good
Cochran & Mays, 2015	2008 General Social Survey (GSS)—National Death Index (NDI) dataset, United States	Adults reporting at least one lifetime sexual partner	Presumptively heterosexual men and women in the dataset (direct)	• Total *n* = 17,886 • Deaths = 3,304	• All-cause • Intentional self-harm/suicide • Breast cancer **•** HIV	**Among sexual minority men** **∅ all-cause** (aHR = 1.04 [0.82–1.31]) **∅ intentional self-harm** (aHR = 0.40 [0.05–3.05]) **∅ HIV-related** (aHR = 1.29 [0.47–3.51]) **∅ other conditions** (aHR = 1.06 [0.83 = 1.36]) **Among sexual minority women:** **⇑ intentional self-harm** (aHR = 6.28 [1.45–27.22]) **∅ all-cause** (aHR = 1.06 [0.78–1.43]) **∅ other conditions** (aHR = 0.96 [0.69–1.33]) **∅ breast cancer** (aHR = 1.82 [0.63–5.32])	Good
Cochran, Bjorkenstam & Mays, 2016	National Death Index, linked to National Health and Nutrition Examination Survey (NHANES), United States	Individuals in NHANES/NDI file who report sexual orientation or sexual behavior	Exclusively heterosexual individuals in NHANES National Death Index File (direct)	• Total *n* = 15,564 • Deaths = 338	**•** All-cause	**⇑ all-cause for sexual minority people** (aHR = 2.02 [1.31–3.11]) **∅ all-cause for sexual minority women** (aHR = 1.54 [0.70–3.36]) **⇑ all-cause for sexual minority men** (aHR = 2.45 [1.42–4.26]) **∅ all-cause for sexual minority men, adjusted for HIV** (aHR = 1.60 [0.69–3.70])	Good
Davis et al., 2017	Interviews with people who inject drugs & Colorado Department of Public Health mortality records, United States	People who inject drugs (PWID) living in Denver, CO between 1996–2001	Heterosexual people who inject drugs (direct)	• Total *n* = 2,007 • Deaths = 86	**•** All-cause	**⇑ gay and lesbian PWID** (HR = 2.22 [1.02–4.82]) **∅ bisexual PWID** (HR = 1.12 [0.54–2.34])	Fair
deBlok et al., 2021	Cause of death from Statistics Netherlands, 1972–2018, Netherlands	Adult transgender patients in a gender identity clinic who received hormone treatment	Overall Dutch population of men and women (indirect)	• Total *n* = 4,568 (2927 transgender women, 1641 transgender men) • Deaths = 361 (317 transgender women, 44 transgender men)	**•** All-cause **•** CVD **•** Lung cancer • Infection • Suicide	**Among transgender women,** by comparison to general population women: **⇑ overall mortality** (SMR = 2.8, [2.5–3.1]) **⇑ CVD** (SMR = 2.6,[1.9–3.4]) **⇑ all cancers** (SMR = 1.6, [1.3–2.0]) **⇑ lung cancer** (SMR = 3.1, [2.1–4.2]) **⇑ infection** (SMR = 8.7, [4.7–14.1]) **⇑ HIV-related disease** (SMR = 47.6 [5.8–132.6]) **⇑ non-natural causes of death** (SMR = 6.1 [2.0–8.4]) **⇑ suicide** (SMR = 6.8 [4.1–10.3]) **Among transgender men**, by comparison to general population men: **∅ overall mortality** (SMR = 1.1 [0.8–1.5]) **∅ CVD** (SMR = 0.8 [0.3–1.6]) **∅ all cancers** (SMR = 0.8 [0.4–1.4]) **∅ lung cancer** (SMR = 1.0 [0.2–2.3]) **∅ non-natural causes of death** (SMR = 1.3 [0.5–2.5]) **∅ suicide** (SMR = 1.2 [0.3–3.0]) **! no infection deaths reported**	Good
Dinno, 2017	**•** National Coalition of Anti-Violence Programs data on transgender homicide **•** International Transgender Day of Remembrance data on transgender homicide **•**Mic interviews **•** National Center for Health Statistics Mortality Multiple Cause-of-Death Annual Public Use Records, 2010–2014 United States	Transgender US residents	General population homicide rate–presumed cisgender, intersected with age, gender and race/ethnicity (indirect)	*n* = 69	**•** Homicide	*Reported statistics assume transgender people represent 0.35% of the population **⇓ homicide for transgender people** compared to cisgender people (RR = 0.24 [0.19–0.31]) **⇑ transfeminine Black people age 15–34 homicide** compared to Black cisfeminine people (RR = 4.03 [2.91–5.57]) **⇑ transfeminine Latin@ people age 15–34 homicide** compared to Latin@ cisfeminine people (RR = 2.40 [1.25–4.62]) **⇓ transfeminine Black people age 15–34 homicide** compared to Black cismasculine people (RR = 0.45 [0.32–0.63]) **⇓ transfeminine Latin@ people age 15–34 homicide** compared to Latin@ cismasculine people (RR = 0.35 [0.18–0.68])	Poor
Feigelman, Ploderl, Rosen & Cerel, 2019	**•** General Social Survey (1988 to 2010) **•** Add Health (Waves 1 and 2) United States	GSS: general US adult population who reported same-sex sexual activity Add Health: general adolescent US population who reported same-sex sexual attraction	GSS: people who reported no lifetime same-sex sexual activity (direct); Add health: people who reported different-sex sexual attraction at Waves 1 and Wave 2 (direct)	GSS: • Total *n* = 31,813 • Self-inflicted deaths = 197 Add Health: • Total *n* = 20,538 • Self-inflicted deaths = 197	**•** Suicide	GSS: **⇑ suicide among sexual minority women** (OR = 4.43 [1.01–19.47] for those with past year sexual minority sexual activity; OR = 4.84 [1.06–22.18] for those with sexual minority sexual activity in past 5 years) **∅ suicide among sexual minority men** (OR = 1.43 [0.44–4.59] for those with past year sexual minority sexual activity; OR = 1.51 [.46–4.87] for those with sexual minority sexual activity in past 5 years) Add health: ∅ **suicide among sexual minority men** (OR = 0.47 [0.06–3.54]) ! **suicide rates** could not be calculated for **women** due to lack of observations	Poor
Frisch & Simonsen, 2013	Danish Civil Registration System, Netherlands	Danish individuals registered in same-sex cohabitating relationships or marriages between 1982–2011	Same-sex cohabitation was compared to opposite-sex cohabitation, multi-adult, and single households; Same-sex married was compared to opposite-sex married (direct)	**•** Total *n* = 6.79 million **•** Total deaths = 1,709,850 **•** Total Person years = 122.5 million **•** Same-sex married, 0.2% **•** Same-sex cohabitating, 2.9%	**•** All-cause **•** Suicide **•** CVD **•** Cancer **•** Respiratory disease **•** AIDS **•** Other causes	**Among same-sex married women:** **⇑ all-cause** (HR = 1.89 [1.60–2.23]) **∅ CVD** (HR = 1.32 [0.75–2.33]) **⇑ cancer** (HR = 1.62[1.28–2.05]) **∅ respiratory disease** (HR = 0.85 [0.36–2.05]) **⇑ suicide** (HR = 6.40[3.42–12.00]) **! AIDS mortality** not observed **⇑ other causes** (HR = 1.47[1.08–2.01]) **Among same-sex married men:** **⇑ all-cause** (HR = 1.38 [1.25–1.53]) **∅ CVD** (HR = 1.23 [0.95–1.35]) **∅ cancer (**HR = 1.12 [0.92–1.35]) **∅ respiratory disease** (HR = 1.12 [0.73–1.74]) **⇑ suicide** (HR = 4.09[2.73–6.12]) **⇑ AIDS** (HR = 356[223–567]). **⇑ other causes** (HR = 1.58[1.32–1.88]) **Among same-sex cohabitating women:** **⇑ all-cause** (HR = 2.04 [1.92–2.18]) **⇑ CVD** (HR = 1.59 [1.39–1.83]) **⇑ cancer** (HR = 1.37[1.22–1.54]) **⇑ respiratory disease** (HR = 2.10[1.74–2.33]) **∅ suicide** (HR = 1.79 [0.99–3.27]), **! AIDS mortality** not observed **⇑ other causes** (HR = 2.48[2.27–2.71]) **Among same-sex cohabitating men:** **⇑ all-cause** (HR = 1.70 [1.62–1.79]) **⇑ CVD** (HR = 1.75 [1.55–1.97]) **∅ cancer** (HR = 1.11[1.00–1.23]) **⇑ respiratory disease** (HR = 2.16[1.80–2.59]) **⇑ suicide** (HR = 3.17[1.60–3.86]), **⇑ AIDS** (HR = 49.8[30.4–81.6]) **⇑ other causes** (HR = 2.79 [2.59–3.01])	Good
Hughes et al., 2022a	Optum Clinformatics Data Mart Database, 2011 to 2019, United States	Transgender enrollees using claims related to gender-affirming care	10% random sample of cisgender enrollees (direct)	• Total *n* = 2,876,073 • Transgender *n* = 18,033	**•** All-cause	**Among Black trans-feminine/nonbinary people:** **⇑** risk of mortality vs. Black cisgender men (SMR = 2.38 [1.29–5.22]) **⇑** risk of mortality vs. Black cisgender women (SMR = 3.3 [1.81–7.31]) **Among white trans-feminine/nonbinary people:** **⇑ risk of mortality** vs. white cisgender men (SMR = 1.98 [1.43–3.11]) **⇑ risk of mortality** vs. white cisgender women (SMR = 2.56 [1.85–4.03]) **Among Black trans-masculine/nonbinary people:** **∅ risk of mortality** vs. Black cisgender men (SMR = 0.86 [0.39–2.62]) **∅ risk of mortality** vs. Black cisgender women (SMR = 1.21 [0.55–3.66]) **Among white trans-masculine/nonbinary people:** **∅ risk of mortality** vs. white cisgender men (SMR = 0.93 [0.60–3.44]) **∅ risk of mortality** vs. white cisgender women (SMR = 1.27 [0.83–4.71])	Good
Hughes et al., 2022b	Optum Clinformatics Data Mart Database, 2011 to 2019, United States	Transgender enrollees using claims related to gender-affirming care	Non-transgender enrollees (direct)	• Total *n* = 4,174,957 • Transgender *n* = 29,758 (58,525 person- years)	**•** All-cause	**Among transgender people overall:** **⇑ mortality risk** vs. cisgender (SMR = 1.80 [1.67–1.93]) **Among trans-feminine/non-binary people:** **⇑ mortality risk** vs. non-trans males (SMR = 1.89 [1.57–2.27]) **⇑ mortality risk** vs. non-trans females (SMR = 2.57 [2.13–3.10]) **Among trans-masculine/non-binary people:** **⇓ mortality risk** vs. non-trans males (SMR = 0.64 [0.52–0.80]) **mortality risk** vs. non-trans females (SMR = 0.87 [0.70–1.08]) **Among unclassified trans people:** **⇑ mortality risk** vs. non-trans males (SMR = 1.93 [1.77–2.10]) **⇑ mortality risk** vs. non-trans females (SMR = 2.45 [2.25–2.67])	Good
Jackson et al., 2023	UK’s Clinical Practice Research Datalink GOLD and Aurum databases, United Kingdom	Patients in about 22% of primary care practices across the UK between 1988 and 2019	Cisgender men and cisgender women primary care patients (direct)	• Total *n* = 139,484 • Transgender *n* = 3,315	**•** All-cause • Infectious and parasitic • Circulatory • Digestive • Nervous system • Respiratory •Endocrine, nutritional, metabolic • External cause • Mental and behavioral • Neoplasm	**Among transfeminine people** vs. cisgender men: **⇑ overall mortality** (MRR = 1.34 [1.06–1.68]) **⇑ cause not elsewhere classified** (MRR = 4.87 [1.71–13.89]) **∅ infectious and parasitic diseases** (MRR = 1.82 [0.41–8.01] **∅ circulatory** (MRR = 1.03 [0.66–1.59]) **∅ digestive** (MRR = 1.15 [0.50–2.66]) **∅ nervous system** (MRR = 0.78 [0.21–2.83]) **∅ respiratory** (MRR = 0.94 [0.47–1.92]) **∅ endocrine, nutritional and metabolic** (MRR = 1.81 [0.59–5.53]) **∅ external causes** (MRR = 1.39 [0.77–2.49]) **∅ mental and behavioral disorders** (MRR = 0.96 [0.34–2.68]) **∅ neoplasms** (MRR = 0.78 [0.50–1.22]) **Among transfeminine people** vs. cisgender women: **⇑ overall mortality** (MRR = 1.60 [1.27–2.01]) **⇑ external causes** (MRR = 1.92 [1.95–3.50] **⇓ neoplasms** (MRR = 0.52 [0.32–0.83]) **∅ infectious and parasitic diseases** (MRR = 1.02 [0.24–4.43]) **∅ circulatory** (MRR = 1.51[0.94–2.42]) **∅ digestive** (MRR = 1.14 [0.50–2.63]) **∅ nervous system (**MRR = 0.54 [0.14–2.07]) **∅ respiratory** (MRR = 0.95 [0.48–1.89]) **∅ endocrine, nutritional and metabolic** (MRR = 2.84 [0.87–9.24]) **∅ mental and behavioral disorders** (MRR = 0.87 [0.32–2.42]) **∅ cause not elsewhere classified** (MRR = 3.49 [0.45–25.79]) **Among transmasculine people** vs. cisgender men: **∅ overall mortality** (MMR = 1.43 [0.87–2.33]) **⇑ cause not elsewhere classified** (MRR = 9.27 [2.93–29.30]) **∅ circulatory** (MRR = 0.83 [0.28–2.46]) **∅ digestive** (MRR = 1.40 [0.16–12.07]) **∅ respiratory** (MRR = 0.89 [0.14–5.74]) **∅ endocrine, nutritional and metabolic** (MRR = 1.91 [0.29–12.60]) **∅ external causes** (MRR = 1.84 [0.81–4.17]) **∅ mental and behavioral disorders** (MRR = 0.93 [0.12–7.02]) **∅ neoplasms** (MRR = 1.06 [0.50–2.27]) **! infectious and parasitic diseases–no cases reported** **! nervous system–no cases reported** **Among transmasculine people** vs. cisgender women: **⇑ overall mortality** (MMR = 1.75 [1.08–2.83]) **⇑ external causes** (MRR = 2.77 [1.15–6.65]) **∅ respiratory** (MRR = 0.98 [0.16–5.90]) **∅ endocrine, nutritional and metabolic** (MRR = 3.11 [0.46–20.90]) **∅ mental and behavioral disorders** (MRR = 0.72 [0.10–5.31]) **∅ neoplasms** (MRR = 0.71 [0.32–1.56]) **∅ cause not elsewhere classified** (MRR = 6.83 [0.88–52.74]) **! infectious and parasitic diseases–no cases reported** **! nervous system–no cases reported**	Good
Laughney & Eliason, 2022	National Health and Nutrition Examination Survey; National Death Index, United States	Survey respondents from 1999–2014	Non-sexual minority survey respondents (direct)	**•** Total *n =* **•** Sexual minority *n* = 1,580	**•** All-cause	**⇑ mortality risk for sexual minority women** vs. non-sexual minority women (aHR = 2.0 [1.3–3.2]) **∅ mortality risk for sexual minority men** vs. non-sexual minority men (aHR = 0.9 [0.5–1.4])	Good
Lehavot et al., 2016	Women’s Health Initiative Study: 21 years of observational study and clinical trials, United States	Postmenopausal women	Sexual minority veterans vs sexual minority non-veterans vs heterosexual veterans vs heterosexual non-veterans (direct)	**•** Total *n =* 137,639 **•** Sexual minority *n* = 1,884	**•** All-cause **•** Cancer **•** CVD	**Among sexual minority women:** **⇑ all-cause** (aHR = 1.20 [1.07–1.36]) **⇑ cancer** (aHR = 1.25 [1.03–1.51]) **∅ CVD** (aHR = 1.17 [0.92–1.50]) **Among sexual minority women veterans:** **∅ all-cause** (aHR = 1.03 [0.73–1.47]) **⇑ cancer** (aHR = 1.70 [1.01–2.85]) **∅ CVD** (aHR = 0.65 [0.30–1.43])	Good
Lindstrom & Rosvall, 2020	Scania Public health survey in 2008, Sweden	Survey responses from stratified sample of the official register population aged 18–80 in Scania	Heterosexual public health survey respondents (direct)	**•** Total *n =* 25,071	**•** All-cause	**Among sexual minority men** vs. heterosexual men: **⇑ mortality** for bisexual men (aHRR = 1.91 [1.10–3.30]) **∅ mortality** for homosexual men (aHRR = 0.85 [0.38–1.90]) **∅ mortality** for “other” men (aHRR = 1.28 [0.84–1.95]) **Among sexual minority women** vs. heterosexual women: **⇑ mortality** for bisexual women (aHRR = 3.18 [1.64–6.18]) **⇑ mortality** for “other” women (aHRR = 2.32 [1.47–3.67]) **∅ mortality** for homosexual women (aHRR = 2.15 [0.89–5.19])	Good
Mathy, Cochran, Olsen & Mays, 2011	Danish National Board of Health 1990–2001 and census data from Statistics Denmark, Denmark	Adults with same-sex registered partnerships	Adults presumed heterosexual (direct)	**•** Total suicides 11,200	**•** Suicide	**⇑ suicide for men in current or former same-sex registered partnership** (IRR = 8.19 [5.48–12.24]) **∅ suicide for women in same-sex registered partnership** (IRR = 1.65 [0.74–3.68])	Good
Mize & Shackelford, 2008	U.S. Federal Bureau of Investigation Supplementary Homicide Reports (SHR); Census data, United States	Perpetrators and victims of intimate partner homicide	Other intimate partner homicides in the SHR database (direct)	**•** Total *n* = 51,007 **•** Total SGM deaths = 1,092	**•** Intimate partner homicide	**⇑ intimate partner homicide among gay couples** (63.72/million/annum vs. 21.25/ million/annum for heterosexual couples) **⇓ intimate partner homicide among lesbian couples** (9.07/million/annum vs. 21.25/million /annum for heterosexual couples)	Poor
Moore et al., 2023	National Health and Nutrition Examination Survey; National Death Index, United States	Representative sample of non-institutionalized US residents, 2001–2010	Heterosexual NHANES respondents (direct)	**•** Total *n* = 12,470	**•** Cancer-related	**⇑ cancer-related mortality** for sexual minority people with high allostatic load vs. heterosexual people with high allostatic load (HR = 2.26 [1.33–3.84]) ∅ **cancer-related mortality** for sexual minority people with low allostatic load vs. heterosexual people with low allostatic load (HR = 0.58 [0.22–1.54])	Good
O’Driscoll et al., 2001	Death certificates/public health records, United States	Cohort of injection drug users (IDUs)	Heterosexual IDUs who died of accidental fatal drug overdose (direct)	**•** Total *n* = 2,849 **•** Total deaths = 72	**•** Fatal drug overdose	**⇑ accidental drug overdose** among **bisexual** IDUs (adjusted RR = 4.86 [2.30–13.20]) **∅ accidental drug overdose** among **gay** IDUs (adjusted RR = 3.54 [0.46–26.9])	Poor/Fair*
Salway et al., 2022	Canadian Community Health Survey; Canadian Mortality Database, Canada	Canadian adults, ages 18–59, 2000–2017	Heterosexual survey respondents (direct)	• Total *n* = 442,260 • 2.4% LGB	**•** All-cause **•** Cancer • Heart disease • Accidents • HIV • Suicide	**Among sexual minority people:** **⇑ all-cause mortality** (uHR = 1.28 [1.06–1.55]) **⇑ heart disease mortality** (uHR = 1.53 [1.03–2.29]) **⇑ accident mortality** (uHR = 1.97 [1.01–3.86]) **∅ cancer mortality** (uHR = 0.86 [0.60–1.25]) **∅ suicide mortality** (uHR = 2.22 [0.93–5.30]) **! HIV mortality estimates not reported**	Good
van Kesteren, Asscheman, Megens & Gooren, 1997	Patient files from Dutch clinic between 1975–1994, Netherlands	People treated with cross-sex hormones	General population statistics, Netherlands Central Bureau of Statistics (indirect)	**•** Total *n* = 1,109 **•** Total deaths = 41	**•** All-cause **•** Suicide **•** Homicide **•** Accidental drowning **•** Deaths of unknown cause **•** Myocardial infarction **•** COPD **•** Pancreatitis **•** AIDS **•** Malignancies	**Among transgender women:** **⇑ suicide** (SMR = 9.29 [4.94–15.88]) **⇑ AIDS** (SMR = 6.0 [1.24–17.53]) **∅ all-cause (**SMR = 0.77 [0.55–1.05]) **∅ homicide** (SMR = 5.0 [0.13–27.86]) **∅ unknown cause** (SMR = 1.67 [0.45–4.27]) **∅ myocardial infarction** (SMR = 0.71 [0.26–1.55]) **∅ COPD** (SMR = 0.87 [0.11–3.14]) **∅ pancreatitis** (SMR = 0.59 [0.01–3.28]) **∅ malignancies** (total SMR = 0.46 [0.20–0.91]; pulmonary SMR = 0.47 [0.10–1.37]; gastric SMR = 0.91 [0.02–5.07]; leukemia SMR = 2.0 [0.05–11.14]) **! accidental drowning, glioblastoma, and meningioma deaths effect sizes not reported** **Among transgender men:** **∅ all-cause** **∅ malignancies** (SMR = 11.49 [0.29–64.05]) **∅ gastric bleeding** (SMR = 5.0 [0.13–27.96])	Good
**Studies reporting no evidence of elevated mortality among SGM**							
Ndimbie et al., 1994	Pitt Men’s Study, which is a component of the Multicenter AIDS Cohort Study (MACS), United States	MSM who were either HIV positive or negative	General population of Allegheny County, Pennsylvania (indirect)	**•** Total *n* = 1,115 **•** Total deaths = 90 **•** Total unexpected deaths = 6	**•** Suicide **•**Homicide **•** Sudden unexpected deaths	**∅ MSM suicide** (No effect size reported) **∅ MSM without AIDS sudden unexpected death rate** (38%) **similar to general population** (45%) **! no homicides observed** **! no sudden unexpected deaths observed among AIDS patients**	Poor

⇑ indicates that SGM group displays significantly higher mortality; ⇓ indicates that SGM group displays significantly lower mortality; ∅ indicates no statistically significant difference in mortality between SGM and comparison group;! indicates insufficient information for analysis; “QA” = quality assessment; * indicates disagreement between raters on Quality Assessment

### Quality assessment

Two study team members (second and senior authors) used the National Institute of Health’s Quality Assessment Tool for Observational Cohort and Cross-Sectional Studies to independently assess the quality of the sampled studies [49]. The raters grouped their ratings into three qualitative groups (Good, Fair, and Poor) and then compared their ratings, achieving 96.55% agreement. Ratings are reported in Table 3.

## Results

Forty-three studies were identified that met study inclusion criteria**.** However, there may be additional relevant studies written in languages other than English, published in journals that are not indexed in the databases we included in our search, or disseminated in non-peer-reviewed distribution channels (e.g., grey literature).

Most reviewed studies came from the United States (n = 27) and the remaining studies originated in the Netherlands (n = 5), Sweden (n = 3), Denmark (n = 4), the United Kingdom (n = 2) and Canada (n = 1); one study included data from both Sweden and Denmark. The earliest reported data among the studies was collected beginning in 1967 [50]. Most studies examining SGM mortality were published within the past 15 years (81.4%), highlighting the relative recency of research on mortality for this population. Of the 43 studies reviewed, 62.8% (n = 27) examined all-cause mortality, 53.5% (n = 23) examined mortality from suicide/intentional self-harm, and 16.3% (n = 7) examined homicide mortality. Three studies (7.0%) examined causes of death related to drug use, including 1 study (2.3%) which focused exclusively on mortality due to drug overdose. Overall, 14 studies (32.6%) reported consistently higher mortality rates and risk for SGM; 28 studies (65.1%) provided partial support for higher mortality among SGM (i.e., evidence for elevated mortality for some causes of death or some SGM subgroups, but not others); and 1 study (2.3%) found no evidence of higher mortality among SGM. Findings are presented in Tables 2 and 3 and described below. In order to maintain consistency with the information provided in the included studies, study results are described using the language of the original reports where applicable.

### All-cause mortality

Studies examining all-cause mortality reported mixed results. Eight all-cause mortality studies found evidence of consistently higher mortality among SGM [50–57]. The remaining 19 all-cause mortality studies reported partial evidence of higher mortality among SGM [40, 41, 43, 58–73]. For example, a Dutch study found overall greater all-cause mortality for transgender women (standardized mortality ratio [SMR] = 1.51, 95%CI = 1.47–1.55) compared to the general Dutch population [59]. However, that same study showed no differences in all-cause mortality for transgender women over age 65 in age stratified models (SMR = 0.95, 95% CI = 0.86–1.06) and did not find overall greater all-cause mortality for transgender men compared to the general Dutch population (SMR = 1.12, 95% CI = 0.87–1.42) [59]. One study using electronic health record (EHR) data from the U.S. Department of Veterans Affairs (VA) found that transgender patients, overall, had significantly lower risk of all-cause mortality compared to a matched cisgender patient group (aHR = 0.90, 95% CI = 0.84–0.97) after adjusting for age, race/ethnicity, marital status, and sex [61]. However, in age-stratified models, transgender patients age 40–64 had significantly lower risk of all-cause mortality whereas transgender patients aged 65 years and older had a nearly 40% increased risk of all-cause mortality compared to cisgender patients [61].

### Cause-specific results

Studies reported evidence of higher mortality due to HIV/AIDS [54, 62, 73], unknown cause [58, 59], “unnatural” cause [50], and illicit drug use [59]. In a cohort study of people who use injection drugs, drug overdose mortality was found to be higher among bisexual, but not gay people who use injection drugs [74]. Most studies examining cancer deaths reported higher rates of cancer mortality for SGM populations overall, including greater lung cancer mortality observed among a sample of transgender women in the Netherlands (SMR = 1.35, 95% CI = 1.14–1.58) [59], elevated cancer mortality among same-sex married women in Denmark (HR = 1.62, 95% CI = 1.28–2.05) [65], and greater cancer mortality among a sample of postmenopausal sexual minority women (HR = 1.25, 95% CI = 1.03–1.51); this effect was further elevated for the sexual minority veterans in the study (HR = 1.70, 95% CI: 1.01–2.85) [70]. Two studies examining mortality specific to breast cancer show conflicting evidence, with 1 study reporting higher breast cancer mortality among women married to or cohabiting with a female relationship partner (aHR = 3.24, 95% CI = 1.01–10.37) [63] and the other study reporting null findings regarding breast cancer mortality rates for women who reported having any female sexual partners in their lifetime (aHR = 1.82. 95% CI = 0.63–5.32) [41]. Studies examining other neoplasms reported heightened neoplasm mortality overall for people who had undergone gender affirming surgery (aHR = 2.1, 95% CI = 1.0–4.6) [51], but did not observe differences in any kind of neoplasm mortality for transgender men who had undergone gender affirming surgery [59].

Cardiovascular/heart disease mortality was reported to be elevated for people who had undergone gender affirming surgery [51], transgender women [59, 73], same-sex cohabiting women and men [65], sexual minority Canadians [72], and sexual minority veterans [56], although some studies also reported null findings for cardiovascular/heart disease mortality rates among same-sex married women and men [65], sexual minority women [70], sexual minority women veterans [70], and transgender men [59]. Four included studies examined SGM mortality due to accidents. Two studies found differential risk for accident mortality among sexual minority people, while the two studies examining accident mortality among gender minority people reported null findings or insufficient data [56, 58, 60, 72]. One study observed sudden unexpected deaths, reporting a null effect (45% of deaths in the comparison group vs. 38% of deaths among gay and bisexual men without HIV), although this finding was based on a very limited number of sudden unexpected deaths in the cohort of gay and bisexual men during the course of that study [75].

One study found evidence that the overall patterns in causes of death among transgender veterans generally do not differ significantly from the patterns in causes of death among the general U.S. population, with diseases of the circulatory system, neoplasms, external causes and diseases of the respiratory system being the four leading causes of death in both groups [43]. However, a few exceptions were reported, with transgender veterans observed to have elevated mortality from digestive system disease (6.8% of deaths among transgender veterans vs. 3.6% of deaths among the general U.S. population) and infectious/parasitic disease (6^th^ leading cause of death among transgender veterans vs. 9^th^ leading cause of death among the general U.S. population), and reduced mortality from diseases of the nervous system (8^th^ leading cause of death among transgender veterans vs. 5^th^ leading cause of death among the general U.S. population).

### Suicide mortality

Studies examining suicide/intentional self-harm mortality for SGM people also displayed mixed results. Five studies examining suicide mortality documented consistently higher suicide mortality for sexual minority people compared to heterosexual people [39, 42, 56, 76, 77]. Transgender people were found to have substantially elevated risk of death by suicide/intentional self-harm compared to cisgender people across 10 studies, although elevated risk was not always observed for both transgender women and transgender men [43, 52, 58–61, 73, 78, 79]. A study comparing transgender and cisgender veterans found that transgender veterans had a nearly threefold risk of suicide mortality (aHR = 2.77, 95% CI = 1.88–4.09) [61]. Elevated suicide mortality risk was also observed for a sample of sexual minority veterans’ whose crude suicide rates were 2.18 times those of non-sexual minorities (82.5 vs. 37.7 per 100,000 person-years) [43]. In a study of suicide death records, LGBT youth aged 12–14 accounted for 24% of suicides in that age group while comprising only 8.9% of the study sample [39].

Five studies of suicide mortality reported partial evidence of higher suicide mortality among SGM, whereby certain SGM subgroups, but not others, displayed higher levels of mortality attributable to suicide in relation to the comparison group [44, 59, 65, 73, 80]. For example, a study reported null findings for sexual minority women (RR = 1.65, 95% CI = 0.74–3.68), but observed greater risk of suicide mortality for sexual minority men (RR = 8.19, 95% CI = 5.48–12.24) [42]. A study of transgender Veterans Health Administration patients reported on differences in risk of death by suicide by several means. After adjusting for demographics and history of depression, transgender VHA patients were at increased risk of suicide by firearm (aHR = 1.83, 95% CI = 1.14–2.94) and self-poisoning (aHR = 3.08, 95% CI = 1.33–7.14) compared to non-transgender VHA patients, but not by hanging, strangulation and suffocation (aHR = 1.31, 95% CI = 0.52–3.27) or other means of self-harm (aHR = 1.05, 95% CI = 0.17–6.53) [79]. Suicide mortality risk was reported to be higher among transgender women than among transgender men in one study (HR = 2.26, 95% CI = 1.06–4.82; 0.8% of transgender women vs. 0.3% of transgender men) [78].

Two other included studies reported no evidence of significant differences in suicide mortality for SGM [72, 75]. However, one of these studies reported constraints on analysis and interpretation of the data due to the rarity of death by suicide among their samples, making it challenging to understand whether null findings are attributable to issues with statistical power or a true null effect [75].

### Homicide mortality

Studies examining homicide mortality for SGM populations also displayed mixed results. One study of VHA patients found consistent evidence of higher homicide mortality among transgender patients in an unadjusted model (HR = 3.67, 95% CI = 1.51–8.91) [61]. However, homicide remained a rare occurrence in both the transgender and cisgender sample, impeding interpretation and generalizability of the findings. Three studies of homicide mortality reported some evidence of higher homicide mortality among SGM, whereby at least one SGM subgroup displayed higher levels of mortality attributable to homicide relative to the comparison group. For example, in a study of intimate partner homicide, gay couples were observed to have a higher rate of intimate partner homicide victimization (63.72 homicides per million per annum) than heterosexual couples (21.25 homicides per million per annum), while lesbian couples were observed to have a lower rate of intimate partner homicide than heterosexual couples (9.07 homicides per million per annum) [81]. In another study, which sought to account for assumed undercounting of transgender people in population estimates, transgender people overall were estimated to have lower risk of homicide mortality in relation to cisgender people, with three-quarters of the RR estimates being below 1.0. The notable exceptions to this pattern were young Black and Latinx transgender women, for whom all RR estimates exceed 1.0, indicating greater risk than the comparison group [82]. One remaining study sought to examine sudden unexpected or violent causes of death, including homicide, but homicide was not found to be a cause of death in any cases and therefore could not be analyzed [75].

#### Measurement of sexual orientation and gender identity in mortality studies

Because operationalization of SOGI can vary across studies, and consequently impact the validity of findings, we also summarized the SOGI measures across included studies.^39^ Of the 28 studies which reported sexual orientation data (62.2%), 12 studies measured sexual orientation via proxy (e.g., same-sex partnership registered with the government), 4 studies measured sexual orientation via self-reported identity only, 4 studies measured sexual orientation via self-reported behavior only, 3 studies measured sexual orientation using both self-reported identity and self-reported behavior and 1 study measured sexual orientation using an unspecified method of self-report. Three studies did not specify how they measured sexual orientation, with two of these studies describing only venue-based recruitment (e.g., “gay venues, bathhouses”), rather than providing information about specifically if or how sexual orientation was assessed, and one study describing neither the method for measuring sexual orientation nor procedures for recruiting sexual minority participants. Of the 17 studies which reported gender identity data (37.8%), all 17 measured gender identity via proxy (e.g., treatment sought for gender dysphoria). Two of the aforementioned studies examined mortality outcomes among both sexual minority and gender diverse people. Table 4 provides an overview of SOGI measurement methods across included studies.

**Table 4:** SOGI measurement in included studies.

Study	SO or GI or both?	Proxy or self-report	Measure of SOGI
Alvarez-Hernandez & Mowbray, 2023	Sexual orientation	Proxy	Informant reports of decedent sexual orientation in NVDRS
Anderson, Marlow & Izugbara, 2023	Both	Proxy	Informant reports of SOGI in NVDRS records, coroner notes and/or law enforcement notes
Asscheman et al, 2011	Gender identity	Proxy	Referral to the outpatient department of university gender clinic
Asscheman, Gooren & Eklund, 1989	Gender identity	Proxy	Presence of reported “problem of gender dysphoria” in outpatient clinic records
Biggs, 2022	Gender identity	Proxy	Referral to pediatric gender clinic
Bjorkenstam et al., 2016	Sexual orientation	Proxy	Registered same-sex partnership or marriage
Blosnich et al., 2014	Gender identity	Proxy	Based on presence of International Classification of Disease (ICD-9) codes related to gender dysphoria in medical records
Blosnich et al., 2021	Gender identity	Proxy	Based on presence of International Classification of Disease (ICD-9 and ICD-10) codes related to gender dysphoria in medical records
Boyer et al., 2021	Gender identity	Proxy	Based on presence of International Classification of Disease (ICD-9 and ICD-10) codes related to transgender identity in medical records
Cochran, Bjorkenstam & Mays, 2016	Sexual orientation	Self-reported identity and/or behavior	Self-reported identity and sex of lifetime sexual partners
Cochran & Mays, 2011	Sexual orientation	Self-reported behavior	Genders of lifetime sexual partners
Cochran & Mays, 2012	Sexual orientation	Proxy	Gender of relationship partner, provided the partner resides within the household
Cochran & Mays, 2015	Sexual orientation	Self-reported behavior	Sex of sexual partners in lifetime or in the past year
Davis et al., 2017	Sexual orientation	Not specified	Not specified
deBlok et al., 2021	Gender identity	Proxy	Previous visit at university gender identity clinic
Dhejne et al., 2011	Gender identity	Proxy	At least one inpatient diagnosis of gender identity disorder without concomitant psychiatric diagnosis and at least one discrepancy between gender variables in selected registries
Dinno 2017	Gender identity	Proxy	Identity stated in media reports
Erlangsen et al., 2020	Sexual orientation	Proxy	Registered same-sex marriage
Erlangsen et al., 2023	Gender identity	Proxy	Based on transgender-related diagnostic codes, recorded legal change of gender, or both
Everett, Wall, Shea & Hughes, 2021	Sexual orientation	Self-reported identity	Presumed sexual minority identity due to enrollment in longitudinal study of sexual minority women which required prior self-report of minoritized sexual identity
Feigelman, Ploderl, Rosen & Cerel, 2019	Sexual orientation	Self-reported behavior	Sex of partners in both the past 12 months and the past 5 years or same-sex romantic attraction
Frisch & Brønnum-Hansen, 2009	Sexual orientation	Proxy	Registered same-sex marriage
Frisch & Simonsen, 2013	Sexual orientation	Proxy	Current or former registered same-sex marriage
Hughes et al., 2022a	Gender identity	Proxy	Based on presence of International Classification of Disease (ICD-9 and ICD-10) codes specific to trans individuals in insurance claims
Hughes et al., 2022b	Gender identity	Proxy	Based on presence of International Classification of Disease (ICD-9 and ICD-10) codes specific to trans individuals in insurance claims
Jackson et al., 2023	Gender identity	Proxy	Diagnosis codes for gender incongruence
Koblin et al., 1992	Sexual orientation	Not specified	Not specified–report only recruitment from clinics serving homosexual men, gay organizations, bathhouses, and residence in areas of New York City where large numbers of homosexual men lived during the study period
Laughney & Eliason, 2022	Sexual orientation	Self-reported identity and/or behavior	History of same-sex sexual partners or identification as homosexual/gay, homosexual/lesbian, or bisexual
Lehavot et al., 2016	Sexual orientation	Self-reported behavior	Sex of sexual partners over adult lifetime
Lindström & Rosvall, 2020	Sexual orientation	Self-reported identity	Self-identification as heterosexual, bisexual, homosexual, or other
Lynch et al., 2020	Sexual orientation	Proxy	Based on presence of International Classification of Disease (ICD-9 and ICD-10) codes related to sexual orientation in medical records and clinical notes
Lynch et al., 2022	Sexual orientation	Proxy	Documentation of minoritized sexual orientation within text or administrative codes in medical records
Martin, Cloninger, Guze & Clayton, 1985	Sexual orientation	Proxy	Index diagnosis of homosexuality
Mathy, Cochran, Olsen & Mays, 2011	Sexual orientation	Proxy	Current or past same-sex registered domestic partnership
Mize & Shackelford, 2008	Sexual orientation	Proxy	Recorded as homosexual relationship in homicide database
Moore et al., 2023	Sexual orientation	Self-reported identity and/or behavior	Self-identification as heterosexual or straight, homosexual or gay, or bisexual, or indication of positive lifetime histories of same-sex sexual partners
Ndimbie et al., 1994	Sexual orientation	Self-report–non-specific	Presumed sexual minority identity due to prior enrollment in epidemiologic study with homosexual and bisexual male subjects
O’Driscoll et al., 2001	Sexual orientation	Self-reported identity	Self-identification as sexual minority
Passaro et al., 2019	Sexual orientation	Not specified	Not specified–report only recruitment from community venues serving gay and bisexual men and media outlets
Ream, 2019	Both	Proxy	NVDRS indicators for sex, sexual orientation, and transgender status
Salway et al., 2022	Sexual orientation	Self-reported identity	Self-identification as heterosexual, homosexual or bisexual
van Kesteren, Asscheman, Megens & Gooren, 1997	Gender identity	Proxy	Presence of reported “problem of gender dysphoria” in outpatient clinic records
Wiepjes et al., 2020	Gender identity	Proxy	Previous visit to gender dysphoria clinic

## Discussion

Public health policy and intervention decision-making depends on the availability of accurate vital statistics including mortality data. Yet research on disparities in SGM mortality remains scant, largely due to still limited progress in adoption of SOGI measures in public health surveillance systems. The current study sought to review mortality studies from the past 5 decades to begin to quantify SGM mortality risk.

In total, we identified just 43 studies that met review criteria representing a wide historical period from data collected 1967 onwards. Findings from this systematic review illustrate that, even with limited data and significant heterogeneity of recording of SOGI information in mortality studies, there seemed concordance in aggregate-level analyses showing greater all-cause mortality for SGM than non-SGM populations. Attempts at disaggregated analysis (e.g., transgender men, transgender women) yielded less consistent findings, likely due to small sample sizes that reduced statistical power. Of the 43 studies, 42 indicated higher SGM mortality for at least one cause of death; however, of those 43 studies, 28 studies included null findings for other causes of death and/or included findings that some SGM groups had reduced mortality risk for at least one cause of death (e.g., intimate partner homicide among lesbian couples, all-cause mortality among transgender patients aged 40–64) [61, 81]. Across studies, notable variation in methods of SOGI measurement, use of SOGI proxies, mortality risk measurement, and data sources hampered study quality.

The operationalization of SOGI measures continues to vex health equity researchers, with the National Academies of Science, Engineering, and Medicine (NASEM) issuing its inaugural attempt in 2022 at guiding data collection in the U.S. [83]. However, this guidance does not include the particular data collection used in mortality surveillance. Efforts have been underway to develop and test training for medicolegal death investigators to include SOGI as standard elements in their investigations of external causes of death (e.g., unintentional and intentional injuries) [37, 84]. However, natural causes of death do not frequently involve investigation or autopsies, thus it is unclear how best to assure documentation of SOGI in case of non-violent deaths, which constitute most deaths in the U.S. Although the NVDRS includes standardized SOGI fields, approximately 4 of 5 cases are missing SOGI data [37]. Consequently, the piecemeal fashion of SOGI definitions revealed in this review underscore the need for efforts to guide data collection in the unique context of mortality. Moreover, the disunity of definitions across studies diminished our confidence in applying meta-analysis because of the risks of population misclassification across studies (e.g., relying solely on data from married/partnered couples, medical record data that defined transgender status through ICD codes).

Expanding on this latter point, with the lack of systematic, consistent, and valid SOGI measures in mortality surveillance, studies included in the current review relied on a variety of SOGI proxies, including same-sex marriage, domestic partnership, and cohabitation, same-sex sexual behavior, and clinical histories of gender dysphoria diagnosis or gender affirming hormone therapy. These proxies overlook unmarried, single, and clinically unengaged SGM people and may result in assuming identity from behavior, risking erasure of bisexual and nonbinary groups, SGM people who lack healthcare access, and people who are not “out” as SGM. In fact, most studies that met inclusion criteria for this review focused on gay and lesbian mortality, and many of those studies relied on same-sex marriage and domestic partnership as proxy measures of sexual orientation. The use of such proxies may help explain why only a few studies in this review specifically provided bisexual mortality statistics, despite decades of research indicating that bisexual people are uniquely burdened by health disparities when compared to gay and lesbian people [85]. Most of the studies of transgender mortality relied on clinical cohorts using proxies for study inclusion, such as ICD-9 diagnostic codes or cross-sex hormone treatment. Such methods exclude non-clinically-engaged transgender and nonbinary people. For example, in the U.S. upwards of 33% of transgender people, generally, and 69% of nonbinary people, specifically, are not enrolled gender-related clinical care, such that a reliance on clinical care for study enrollment may specifically undercount nonbinary populations [86].

Most of the studies in this review focused on all-cause and suicide mortality. Far fewer studies examined specific causes like cancer, drug overdose, and homicide. Future research should also explore links between established disparities in smoking and potential increased cancer and cardiovascular disease mortality among SGM people [87]. Given the recent and ongoing opioid epidemic in the U.S, studies of mortality disparity by overdose may be useful to guiding substance use intervention with SGM people. For example, a recent study of VA patient records found that transgender patients had over twice the risk of opioid poisoning than cisgender patients [88]. Furthermore, studies on homicide may be particularly relevant and needed considering recent grassroots movements to combat hate violence and homicide of trans women of color, specifically [89–92]. Transgender individuals have greater risks of intimate partner violence (IPV) than their cisgender peers, a finding replicated across geography, including Haiti, Australia, China, and Brazil. IPV is a major risk factor for premature mortality, especially intimate partner homicide [93–97].

In addition to limitations in assessment of SOGI information across studies and limited scope of causes of death, several other limitations should be considered when interpreting conclusions of this systematic review. Notably, the HIV/AIDS epidemic has been a major cause of mortality for gay and bisexual men and transgender people for decades. This review excluded studies entirely focused on HIV/AIDS clinical and cohort samples to reduce population-level bias in mortality data and potential confounding effects of historical events related to HIV/AIDS treatment and mortality (e.g., introduction of antiretroviral therapy). Because studies on HIV/AIDS cohorts were excluded, the present study is limited in its ability to illuminate the impacts of HIV/AIDS to patterns of mortality among SGM people, although all-cause mortality may be elevated for SGM people with HIV/AIDS. For example, in addition to reductions in life expectancy related to HIV/AIDS among these cohorts, research suggests that disparities in other causes of death (e.g., suicide deaths) may be exacerbated among people living with HIV/AIDS [98, 99].

The results of the review were limited to its search terms, databases (APA, CINAHL, Health Source, Health Policy Reference Center, LGBTQ+ Source, Social Sciences Full Text, MedLine and PubMed), the use of controlled vocabularies over text word searching and each databases’ indexing procedures; it is possible that relevant studies may have eluded the search due to not matching search terms, being written in languages other than English, being published in journals which were not indexed in our chosen databases, or not being peer-reviewed. Additional sources of data (e.g., grey literature) may provide additional insight into mortality disparities for SGM people and should be considered in future research.

Although the review aimed to achieve a global synthesis of SGM mortality data, only studies from North American and European nations met criteria for inclusion. Although disparities in mortality for SGM people are a global issue, we are unable to comment on global patterns due to the absence of comparative mortality studies from South America, Asia, Africa and Oceania in this review. Recent studies suggest that researchers are leading efforts toward understanding SGM mortality in nations where population data on SOGI are unavailable [100–102]. A few studies from nations without population SOGI data were coded as part of our systematic review, but were ultimately excluded due to not meeting inclusion criteria for study design (e.g., online media reports, case studies) or for lacking a non-SGM comparator. However, these studies provide valuable insight about cultural differences which may be associated with mortality risk for SGM people, including each nation’s social norms and legal protections (e.g., social exclusion and homicide of transgender people in Pakistan) [102]. For example, in their study of Tokyo suicides from 2009–2018, Sakai and colleagues compared correlates of suicide, such as means of suicide (e.g., hanging, fall, poison) and mental health diagnoses, for LGBT and non-LGBT populations. Though excluded from this study for not providing overall suicide rate outcomes for LGBT and non-LGBT populations, this work emphasized population differences in predictors of and methods by which suicide occurs in Tokyo that may can inform future public health efforts and research [101]. In an example from India, which was excluded for not reporting a mortality rate outcome, researchers identified that news reports of LGBTQI+ suicide largely covered transgender suicides, often included derogatory terminology (especially when written in a local language), and rarely included educational information on suicide prevention [100]. In addition to limited representation of SGM mortality data globally, methods in the sampled literature notably differed by nationality. Many Northern European studies benefitted from national and civil registries that collected SOGI data, therefore having a greater impact on our findings compared to studies from nations that do not collect SOGI in administrative data.

Although a few included studies examined differential outcomes based on racial and ethnic identity, intersectional approaches were not largely adopted across studies [66, 82]. Therefore, it remains difficult to ascertain how mortality metrics may differ when accounting for multiple marginalization, including marginalization due to diverse racial and ethnic identities and marginalization due to plurisexual identities (e.g., bisexual+). Examining mortality disparities through an intersectional lens is an important direction for exploration in future research. Additionally, several US studies utilized samples of SGM veterans, who may not be representative of non-veteran SGM due to potential additional mortality risks conferred by stressors unique to veteran populations [56]. Each of these limitations may impact generalizability of the findings to other SGM people.

Clarifying the findings from this systematic review requires strengthening mortality surveillance to include SOGI information about decedents on a global scale, which could be achieved in several ways. Enhancing collection of SOGI information across public health surveillance systems, including governmental and municipal health surveys, medical records and hospital systems, and administrative databases, creates opportunities to merge those data with mortality records. Continuing non-collection of SOGI data inhibits meaningful efforts to address social determinants of health through public health policy and programming [103]. Indeed, recent policy efforts in the U.S. (e.g., LGBTQI+ Data Inclusion Act) have aimed to address this need in federal surveys. Researchers in New Zealand constructed a conceptual plan for integration of sexual orientation into governmental statistics systems, presumably including mortality data, and highlighted the importance of cultural relevance of data collection, such as terms and phrases for sexual orientation concepts among indigenous communities [104]. Relatedly, funding for implementation and evaluation of data collection methods is also of great importance because SOGI may have differential response based on demographics (e.g., native/preferred language) and because the constructs of sexual orientation and gender identity are temporally specific and develop over time [105].

In sum, this review offers tentative support that SGM people face unique increased mortality risk compared to heterosexual and cisgender people. Most studies supported the existence of all-cause or cause-specific mortality disparities burdening SGM people. In many studies, one or more subgroups of SGM populations exhibited higher risk of all-cause or cause-specific mortality; future research can examine subsample-specific and cause-specific mortality risks. Further research is needed to work towards harmonizing operationalization and measurement of SOGI to enable meta-analysis, specifically attending to the threats of misclassification and non-representativeness of SGM groups across research designs. Study findings strongly indicate the need to increase rigor and harmonize measures to facilitate more accurate population SOGI mortality assessment. Clear identification of mortality disparities and understanding of mortality risk associated with certain causes of death (e.g., lung cancer) among SGM people is needed to inform targeted public health prevention and medical intervention efforts with SGM people. Studies in this review indicate the need for more population-level research on all-cause and cause-specific SGM mortality risk, with a focus on suicide, homicide, cardiovascular disease, drug overdose, infectious and parasitic diseases, and cancer. More notably, to improve population health equity, findings indicate the need for SOGI data collection in public health surveillance and further research on bisexual, transgender, and nonbinary population health.

## Supporting information

S1 Table Search strategy. (DOCX)

S2 Table PRISMA checklist. (DOC)
