# Supplementary material for: Mortality among sexual and gender minority populations: A systematic review
Source: PLoS One. 2025 Feb 3;20(2):e0307688. doi: 10.1371/journal.pone.0307688 (PMC12140117; doi:10.1371/journal.pone.0307688)
Supplement: S1 Table — (DOCX) [file pone.0307688.s001.docx]

**S1 Table.** Search strategy.

| Database(s) | Dates of searches | Population search terms | Boolean operator | Outcome search terms |
| --- | --- | --- | --- | --- |
| APA (PsycArticles, PsycBooks, PsycINFO) | 06/29/2021  07/18/2023 | LGBTQ OR male homosexuality OR lesbianism OR bisexuality OR transgender OR sexual orientation OR homosexuality OR sexual minority groups OR same-sex couples OR same-sex marriage OR sexual minority groups OR transsexualism OR transvestism OR same sex intercourse OR gender nonconforming OR sex and gender measures OR gender identity | AND | death OR mortality risk OR mortality rate OR suicide OR homicide OR autopsy |
| CINAHL | 06/29/2021  07/18/2023 | LGBTQ+ persons OR gay persons OR gay men OR lesbians OR bisexuals OR transgender persons OR sexual and gender minorities OR transsexuals OR transsexualism OR men who have sex with men OR gender nonconformity OR gender identity | AND | death OR mortality OR suicide OR homicide OR autopsy |
| Health Policy Reference Center | 06/29/2021  07/18/2023 | LGBTQ+ people OR gay people OR gay men OR gay identity OR lesbians OR lesbian identity OR bisexuals OR bisexual identity OR transgender people OR transgender identity OR sexual minority men OR sexual minority women OR homosexuality OR same-sex dating OR same-sex marriage OR gay couples OR sexual minorities OR gender non-conforming people OR trans men OR trans women OR transsexuals OR transsexualism OR transgenderists OR transgenderism OR cross-dressers OR men who have sex with men OR women who have sex with women OR gender identity | AND | death OR mortality OR suicide OR homicide OR murder OR dead OR autopsy |
| LGBTQ+ Source | 06/29/2021  07/18/2023 | LGBTQ+ people OR gay people OR gay men OR gay identity OR lesbians OR lesbian identity OR bisexuals OR bisexual identity OR transgender people OR LGBTQ+ people OR homosexuality OR same-sex dating OR same-sex marriage OR sexual minorities OR gender non-conforming people OR trans men OR trans women OR transsexuals OR transsexualism OR transgenderists OR transgenderism OR cross-dressers OR men who have sex with men OR women who have sex with women OR gender identity | AND | death OR mortality OR suicide OR homicide OR murder OR dead OR autopsy |
| Social Sciences Full Text | 06/29/2021  07/18/2023 | LGBTQ+ people OR gay people OR gay men OR gay identity OR lesbians OR lesbian identity OR bisexuality OR transgender people OR trans women OR trans men OR transgenderism OR homosexuality OR same-sex relationships OR sexual minorities OR gender non-conforming people OR cross-dressers OR men who have sex with men OR women who have sex with women OR gender identity | AND | AND death OR mortality OR suicide OR homicide OR murder OR dead OR autopsy |
| Health Source | 06/29/2021  07/18/2023 | LGBTQ+ people OR gay people OR gay men OR gay identity OR lesbians OR lesbian identity OR bisexuals OR transgender people OR homosexuality OR same-sex relationships OR sexual minorities OR gender non-conforming people OR trans men OR trans women OR transsexuals OR transsexualism OR cross-dressing OR men who have sex with men OR women who have sex with women OR gender identity | AND | death OR mortality OR suicide OR homicide OR murder OR dead OR autopsy |
| MEDLINE & PubMed | 06/29/2021  07/18/2023 | Sexual and gender minorities OR homosexuality OR homosexuality, female OR homosexuality, male OR bisexuality OR transgender persons | AND | death OR mortality OR suicide OR suicide, completed OR homicide OR autopsy |
